# Evaluation of an HIV prevention intervention for women living with HIV

**DOI:** 10.1080/09540121.2019.1659910

**Published:** 2019-08-28

**Authors:** Tanesha Griffin Joshua, Weston O. Williams, Shaliondel Benton, Gary Uhl

**Affiliations:** a Centers for Disease Control and Prevention, National Center for HIV/AIDS, Viral Hepatitis, STD, and TB, Division of HIV/AIDS Prevention, Atlanta, GA, USA; b Public Health Analytic Consulting Services, Inc., Hillsborough, NC, USA

**Keywords:** HIV, behavioral intervention, female, safe-sex, condoms

## Abstract

It is estimated that 23% of the adults and adolescents living with HIV in the United States are female. The Division of HIV/AIDS Prevention at the Centers for Disease Control and Prevention (CDC funds evidence-based interventions (EBIs) to reduce HIV risk behaviors, including HIV prevention programs for people living with HIV and their partners. While EBIs have been shown to be effective in controlled research environments, there are limited data on intervention implementation in real-world settings. Women Involved in Life Learning from Other Women (WILLOW) is a four-session small-group intervention that targets heterosexual women aged 18–50 who are living with HIV. This evaluation assessed changes in participants’ HIV knowledge, attitudes, beliefs and risk behaviors. A repeated measures design was used to collect participant risk behaviors at baseline, and again at three and six months post-intervention. Changes in attitudes, beliefs, and risk behaviors were assessed using generalized estimating equations. After participation in WILLOW, participants reported increased HIV knowledge, attitudes and beliefs, being more supportive of condom use, and reduced prevalence of HIV risk behaviors. Findings suggest that the WILLOW intervention can be successfully delivered by community-based organizations to reduce HIV risk behaviors among members of this high-risk population.

## Introduction

The prevention of HIV transmission is a critical component of the national HIV/AIDS strategy (National HIV/AIDS Strategy for the United States: Updated to 2020, 2015). Reducing HIV risk behaviors among persons living with HIV/AIDS (PLWHA) contributes to preventing HIV transmission (Brown & Diclemente, 2011) and is an appropriate use of HIV prevention resources. A recent study used modeling to examine the optimal allocation of HIV prevention funds and found that behavioral interventions remain an important part of prevention funded by the Centers for Disease Control and Prevention (CDC), and those interventions that focused on PLWHA consistently had a lower cost per new case prevented as compared to interventions for non-infected individuals (Yaylali et al., 2018).

In 2016, an estimated 258,000 women in the United States were living with HIV, and more than 18% of the estimated 38,700 new HIV diagnoses in the United States in 2016 were among women (Centers for Disease Control and Prevention, 2019). Despite this, few interventions to reduce HIV risk behaviors among PLWHA have been designed for women (Brown & Diclemente, 2011). Women Involved in Life Learning from Other Women (WILLOW) is one intervention focused on reducing HIV risk among women living with HIV/AIDS.

### Background of WILLOW

WILLOW is an intervention designed to engage and train 18–50 year-old heterosexual women living with HIV/AIDS to reduce HIV transmission-related risk behaviors and sexually transmitted infections (STI) (Wingood et al., 2004). WILLOW consists of four small group sessions, each four hours long for a total of 16 hours (i.e., one cycle). Each session is facilitated by two trained women, at least one being HIV-positive. WILLOW employs a culturally and gender-appropriate approach to provide women with information about healthy relationships, negotiation of safer sexual practices, social support networks, STI/HIV reinfection and proper condom use, and to train women in skills to support safer sexual behavior (“Women Involved in Life Learning from Other Women: A Small Group-level, Social Skills Building and Educational Intervention for Women Living with HIV/AIDS Fact Sheet,” 2009). Women are provided tools to identify unhealthy relationships and taught negotiation skills. WILLOW also focuses on building social networks to empower women living with HIV.

The original WILLOW study was conducted with women living with HIV who were recruited from 7 clinics and health departments in Alabama (Birmingham and Montgomery) and Georgia (Atlanta metro area). Among those recruited and enrolled in the study, 366 women were randomized into one of the two study groups, “sexual risk reduction and social network” or a health promotion comparison. This study found that WILLOW was effective in reducing sex without a condom and chlamydia/gonorrhea and improving knowledge about HIV, condom use self-efficacy, and reducing beliefs that condoms interfere with sex (Wingood et al., 2004).

### Purpose

We sought to evaluate the intervention in practice at community-based organizations (CBOs) funded by the CDC to implement WILLOW. It is important to know if WILLOW, when implemented outside of a research setting, can result in changes found in the original study. Changes in HIV risk behaviors (sex without a condom including various contexts and partner types) and HIV knowledge, attitudes toward condom use, and ability to use condoms among clients attending the sessions at four CDC-funded CBOs were assessed.

## Materials and methods

### Sample and setting

CDC funded CBOs to conduct the Community-based organization Monitoring and Evaluation Project (CMEP) of WILLOW (CMEP-WILLOW) through the PS 10–1003 program announcement (Centers for Disease Control and Prevention, 2010). Four CBOs were selected based upon evaluation experience, organizational capacity, and proposed project plan through a competitive process to receive additional funding to participate in the project for five years. The four selected CBOs are located in New York City, NY (CBO A), Boston, MA (CBO B), Miami, FL (CBO C), and Newark, DE (CBO D).

WILLOW participants were recruited at a variety of settings such as medical clinics, residential substance abuse treatment facilities, community health centers, AIDS service organizations, and in-house agency programs. Participants were also recruited via word-of-mouth and through referrals from WILLOW graduates. All women living with HIV/AIDS who were 18 years or older, knew of their HIV-positive status for at least six months, and signed up to participate in WILLOW during the evaluation project were asked to participate in the evaluation. Participation in CMEP-WILLOW was voluntary for WILLOW participants. All CMEP-WILLOW participants signed an agreement form that detailed the purpose, the risks/benefits of participation, confidentiality and voluntary nature of participation. Incentives including transportation cards, small personal care items, condoms, and gift cards were issued to clients throughout the WILLOW intervention and CMEP-WILLOW.

### Data collection procedures

Evaluation data were collected for all WILLOW cycles conducted between 1 November 2011 and 30 June 2014. CMEP-WILLOW employed a repeated measures design to collect client-level data at baseline (up to thirty days prior to the start of the intervention) and at 90- and 180-days following the last session of the intervention cycle. Data were collected via a computer-assisted self-interview (CASI) using the Questionnaire Development System (QDS). WILLOW participation attendance, session activities, and client feedback were also collected in QDS.

### Measures and constructed variables

Measures were self-reported and collected using a CASI. CBO staff members were available to answer questions.

### Sociodemographic and relationship variables

Sociodemographic variables collected include:

- Age: Categorized as 18–24,25–34,35–44,45–54, and 55+.
- Race/ethnicity: Analyzed categories included Hispanic/Latino, non-Hispanic white, non-Hispanic black/African American, and non-Hispanic other race (American Indian or Alaskan Native, Asian, Native-Hawaiian/Pacific Islander, or multiple races)
- Educational attainment: Analyzed as a categorical variable, including less than high school diploma, high school diploma/GED, and some college/college degree
- Primary partner: Defined in the survey as someone you have a special emotional connection with, live with, love, and/or call your boyfriend or husband
- Time since first positive test: Calculated using the reported date of the first positive test

### HIV risk behaviors

Participants reported the total number of sex events and the number of sex events when a condom was used in the past three months. Questions also asked about partner attributes and sex contexts, inquiring as to how often condoms were used (never, sometimes, or always). The following variables were constructed for this analysis:

- Any sex without a condom (one or more sex events without a condom) was assessed to evaluate potential decreases in the proportion of women who reported a sex event for which transmission was a risk through sex without a condom. This was also by context of sex and partner attributes, including:
  - Sex without a condom (any) with a partner who: had an HIV-negative or unknown HIV status; were primary/casual partners; were anonymous; were persons who inject drugs (PWID); spent 24 hours or longer in jail/prison; had an STI.
  - Sex without a condom (any) during sex that was: a one-night stand; in exchange (gave/received something); while intoxicated or high on drugs.
- Percent of sex events that were without a condom was assessed to evaluate potential increases in condom use that may not be detected using a dichotomous variable. The number of sex events for which a condom was not used and the number of sex events (as an offset) were used in the statistical analysis to assess changes in this variable.

### Anti-Retroviral Treatment (ART)

Participants reported whether they were prescribed ART (yes/no) and taking ART as directed over the last six months (yes/no). Adherence was defined based upon whether they answered that they had/had not been taking ART as directed.

### HIV knowledge, attitudes, and beliefs

Knowledge, attitudes, and beliefs measures were adapted based upon the measures used in the randomized trial for WILLOW (Wingood et al., 2004), and survey questions are provided in the e-supplement.

- HIV Knowledge: A high score (at least 8/10 correct) from ten true/false questions to evaluate HIV knowledge. Internal consistency was not assessed for the HIV knowledge measure; our assessment focused on whether participants had a high score on these specific questions (i.e., not as an underlying latent construct).
- Attitudes and beliefs toward condoms for partners (Cronbach’s alpha = 0.75): two agree/disagree questions
- Attitudes and beliefs toward condom use for self (Cronbach’s alpha = 0.74): five agree/disagree questions
- Ability to discuss condom use (Cronbach’s alpha = 0.76): seven yes/maybe/no questions
- Ability to use condoms (Cronbach’s alpha = 0.89): nine questions about skills related to condom, with potential responses including “not at all”, “somewhat”, and “very sure”.

All attitudes and beliefs scales were dichotomized at the 25th percentile. The median was explored as a potential point of dichotomy, which has been applied in previous studies, but >50% of the respondents were at the maximum value for each of the scales (Wingood & DiClemente, 1998).

### Data cleaning

Consistency between related variables were checked and one or more consistency recodes were applied for *n* = 88 (3.2%) observations. Observations where no sex without a condom (overall) was reported but sex without a condom with primary and/or casual partners was reported were recoded to having reported sex without a condom (*n* = 73). Individuals who reported that they had sex without a condom (overall) but reported no sex without a condom with primary/non-primary/other partner types were counted as not reporting sex without a condom (*n* = 11). Observations where sex without a condom (overall) was reported, no sex without a condom with primary/non-primary partners was reported, and sex without a condom with another partner type was reported were recoded to set sex without a condom with primary/non-primary to missing (*n* = 4).

### Exclusions

**Figure 1.**
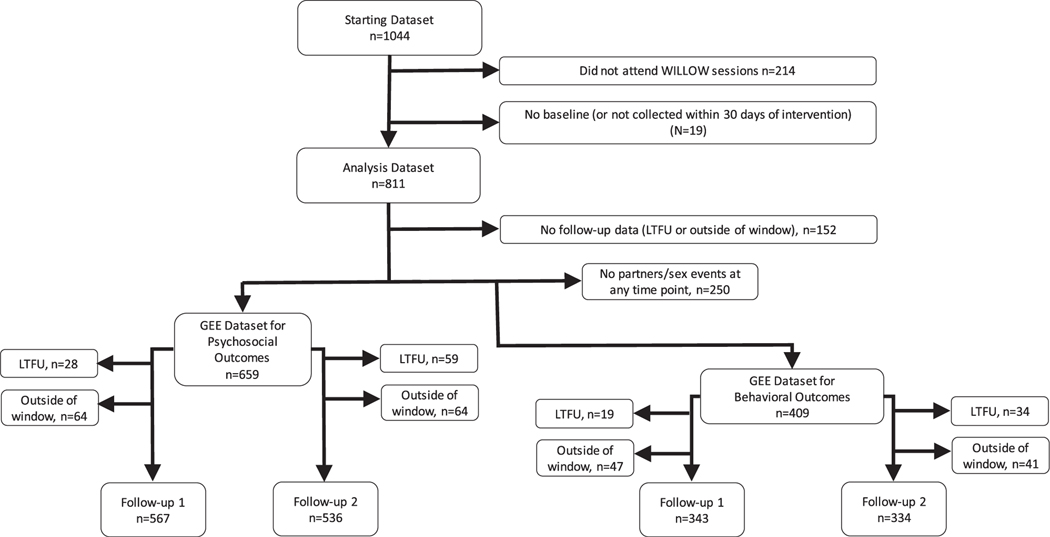
Exclusions applied to analysis dataset.

Data collected within 30 days before the intervention (baseline) or ±2 weeks of the intended follow-up time “window” (90 days for follow-up 1, 180 days for follow-up 2) were included in the analysis. Of *n* = 1,044 individuals for whom data were collected, *n* = 811 (78%) were included in the baseline analysis dataset, and of those individuals, *n* = 659 (81%) were included in the longitudinal analysis of knowledge, attitudes, and beliefs, and *n* = 409 (50%) were included in the longitudinal analysis of HIV risk behavior outcomes (see Figure 1). For longitudinal analyses, participants who provided no follow-up data were also excluded. For HIV risk behavior outcomes, individuals who reported no sex partners/sex events at any time point were also excluded from longitudinal analyses (*n* = 250) to focus on changes in HIV risk behaviors.

Of participants in the analysis sample (*n* = 811), individuals who were excluded due to missing both follow-up visits more frequently reported white (OR = 2.44, 95% Cl 1.08–5.53) or Hispanic/Latino (OR = 2.22, 95% Cl 1.22–4.02) as compared to black/African American race/ethnicity, and were more often diagnosed 3–5 years prior (OR = 2.75, 95% Cl 1.40–5.43) as compared to being diagnosed more than ten years prior. Having a primary partner was associated with lower odds of being excluded (OR = 0.56, 95% Cl 0.33–0.93). With respect to HIV risk behaviors and HIV knowledge, attitudes, and beliefs, only one statistically significant difference was observed; sex without a condom with a primary partner was less frequently reported by those who were excluded (OR = 0.35, 95% Cl 0.12–0.97).

### Analysis

Sample characteristics were summarized using means/standard deviations and percentages. Changes in outcomes at each follow-up, compared to baseline, were assessed using generalized estimating equations (GEE), which account for the lack of independence between repeated observations for individuals in the study (Zeger & Liang, 1986). Adherence to ART in the last six months was assessed by comparing follow-up 2 to baseline (not follow-up 1 to avoid overlap with baseline). Dichotomous outcomes were assessed using logistic models and the percent of sex events that were unprotected was assessed using a negative binomial model with the number of sex events without a condom as the outcome and the log total number of sex events as an offset. Analyses were conducted in SAS 9.3 (Cary, NC). Results are reported for models adjusting for CBO, age, race/ethnicity, educational attainment, time since first testing positive for HIV, and reporting a primary partner.

## Results

### Intervention characteristics and session attendance

Intervention characteristics are described in Table 1. The mean intervention duration was 18.8 hours. The mean number of participants in each cycle 9.9. Most participants (67.6%) attended all four intervention sessions, 11.5% attended some (1–3) intervention sessions, and 20.9% did not participate in any intervention sessions. Session attendance varied by CBO, with CBO B reporting the highest percent completing all sessions (77.8%) and CBO A reporting the lowest (56.2%). Session attendance was not significantly associated with any independent or dependent variables.

### Retention in the evaluation

Of the *n* = 811 individuals included in the analysis dataset, most participants (76.8%) completed both follow-up surveys, 9.9% completed the first follow-up only, 5.2% completed the second follow-up only, and 8.1% did not complete any follow-up surveys. CBO A reported the highest percentage completing both follow-ups (90.6%) and CBO B reported the lowest (71.7%).

### Participant characteristics at baseline

Participant baseline characteristics by CBO and overall are described in Table 2. Most participants were born female (98.6%), and identified as female (98.5%), reported non-Hispanic black/African American race (73.6%), and had never been married (55.2%). Age between 45–54 years was reported by 44.5% of participants and 31.2% had educational attainment of a high school graduate or GED.

### Outcomes at baseline

Among the participants included in the longitudinal analyses, 66.0% scored 80% or higher on Knowledge about HIV at baseline, and 93.2% reported that they were adherent to ART (Table 3). Sex without a condom was reported by 28.7% of the participants at baseline. Sex without a condom was reported by 24% and 5.9% with primary and non-primary partners, respectively. Sex without a condom with a HIV negative partner was reported by 12.6% at baseline, and 5.4% reported sex without a condom with a partner of unknown HIV serostatus.

### Changes in outcomes over time

Significant improvements were observed at both follow-up time points compared to baseline in the percent of sex events that were without a condom, knowledge about HIV, attitudes and beliefs toward condom use (both partner and self), ability to discuss condom use, and ability to use condoms (Table 3). The prevalence of self-reported ART adherence was significantly higher at follow-up 2 as compared to baseline. The odds of reporting sex without a condom overall, with primary partners, with HIV negative partners, and while intoxicated or high on drugs were significantly lower at follow-up 2 as compared to baseline, but not at follow-up 1. Reductions in reporting any sex without a condom with casual and HIV serostatus unknown partners were not statistically significant. Sex without a condom with partners who were anonymous, persons who inject drugs, spent at least 24 hours in jail, had a STI, one-night stands, or exchanged sex money, drugs, or other needs were infrequently reported (all <3%) and were not statistically assessed in the GEE model.

## Discussion

Our findings are consistent with those observed in the randomized control trial for WILLOW, in that we observed significant reductions in several HIV risk behaviors and improvements in HIV knowledge, attitudes, and beliefs after participating in the intervention. These findings support that WILLOW can be successfully implemented in the “real world” by community-based organizations. As in the original research, women who participated in WILLOW were less likely to report sex without a condom six months after participating in the intervention. Significant improvements at 6 months were also noted for HIV knowledge, condom self-efficacy, belief that condoms interfere with sex, and partner barriers to condom use that participants reported.

Some challenges among women living with HIV were encountered during the implementation of WILLOW. CBOs reported that women were frequently unable to meet the intervention and evaluation project requirements due to sickness, lack of child-care, other family responsibilities, and work. WILLOW groups are designed to be completed in the group with which they started, so participation could not be resumed by attending the missed session with another group. Make-up sessions were offered during our implementation of CMEP-WILLOW, which allowed women to continue with their group without re-starting the intervention. The use of make-up sessions may be considered as a potential strategy to encourage successful completion of the intervention.

There are several limitations to this study. Self-reported data may result in social desirability bias when reporting HIV risk behaviors. We collected data using a CASI in attempt to limit this bias. Since randomized sampling procedures were not used, we cannot assume that the sample of clients or CBOs were representative of all clients or CBOs funded by CDC to implement WILLOW. Self-selection to participate may have resulted in a study sample of women who are more alike in attitudes and behaviors. Multiple recruitment methods were employed and women recruited from medical settings may be more likely to participate, as they are already in HIV medical care. Because our study did not include a control group, exposure to factors beyond participation in WILLOW may have influenced changes in outcomes over time. We were not able to meaningfully compare changes in outcomes by differences in exposure to WILLOW because the majority of participants either completed all sessions or none (and those with no sessions did not attend follow-up visits). For some outcomes, such as sex without a condom with a partner who was anonymous, PWID, spent 24 hours in jail, or had a STI, baseline frequencies were very low and we were not able to assess changes after the intervention. CDC did not collect detailed fidelity information assessing, for example, information about the facilitators of the WILLOW sessions. The CBOs did, however, report that the intervention was delivered with fidelity. Nearly a fourth of the participants in the study were lost to follow-up due to relocation, sickness, and death. At the time of the evaluation, the WILLOW intervention did not include messaging about viral suppression and condom use. Nevertheless, some ART adherent participants may not have felt the need to use condoms consistently, as has been described previously e.g., Wilson et al. (2007). The intervention is currently being adapted to include messaging about HIV treatment as prevention.

As funding in the United States for HIV prevention has become tighter, it is even more important to focus prevention efforts in areas of the greatest impact (U.S. Federal Funding for HIV/AIDS: Trends Over Time, 2017). The CDC’s policy to implement high-impact HIV prevention focuses on reaching the right populations in the right areas to increase the impact of HIV prevention efforts (Centers for Disease Control and Prevention, 2011). WILLOW is the only CDC-supported behavioral intervention designed specifically for women living with HIV, making it an important tool that concentrates efforts on a high-risk population. This evaluation of WILLOW confirmed that the intervention, when implemented by these four CDC-funded CBOs, produced improvements that are similar to the original randomized trial. Our findings support the continued implementation of WILLOW to prevent HIV transmission among women living with HIV.

## Supplementary Material

KAB questions

## Figures and Tables

**Table 1.** Intervention characteristics and session attendance by CBO and overall, CMEP-WILLOW, November 2011 - February 2015.

	CBO A	CBO B	CBO C	CBO D	Total

Intervention Cycle Attributes	*N* = 2	*N* = 40	*N* = 35	*N* = 35	*N* = 132
Cycle duration in hours, mean (SD)	20.8 (3.2)	19.9 (0.5)	18.9 (1.5)	16.1 (0.5)	18.8 (2.3)
Participants enrolled in cycle, mean (SD)	15.4(2.8)	8.6 (3.3)	10.6 (2.3)	7.3 (2.9)	9.9 (4.0)
Intervention session attendance^†^	*N* = 249	*N* = 203	*N* = 322	*N* = 251	*N* = 1025
No sessions, %	31.3	7.9	17.4	25.5	20.9
Some sessions (1–3), %	12.5	14.3	7.1	13.9	11.5
All sessions (4), %	56.2	77.8	75.5	60.6	67.6

† lncludes individuals that were excluded for not participating in intervention sessions.

**Table 2.** Participant characteristics by CBO and overall, CMEP-WILLOW, November 2011 - February 2015.

Demographics	CBO A % *n* = 171	CBO B % *n* = 187	CBO C % *n* = 266	CBO D % *n* = 187	Total % *n* = 811

Sex at birth					
Male	4.1	0	1.1	0.5	1.4
Female	95.9	100	98.9	99.5	98.6
Gender identity					
Male	0	0	0	0	0
Female	95.3	100	98.9	99.5	98.5
Transgender- male to female	4.1	0	1.1	0.5	1.4
Transgender- female to male	0.6	0	0	0	0.1
Age group					
18–24	3.5	1.6	6.8	4.3	4.3
25–34	7.0	9.1	11.7	8.0	9.2
35–44	16.4	27.8	19.9	24.1	21.9
45–54	46.8	46.5	40.6	46.0	44.5
55+	26.3	15	21.1	17.6	20.0
Race/ethnicity					
Hispanic/Latino	24.1	19.8	12.5	14.0	17.0
Black/African American, Non-Hispanic	68.2	57.2	84.5	79.6	73.6
White, Non-Hispanic	4.1	18.7	2.3	4.3	6.9
Other, Non-Hispanic	3.5	4.3	0.8	2.2	2.5
Education					
Less than a high school diploma	46.8	36.9	46.6	40.6	43
High school graduate/GED	25.1	32.6	30.1	36.9	31.2
Some college	23.4	23	20.3	20.3	21.6
Bachelor’s degree or higher	4.7	7.5	3.0	2.1	4.2
Marital status					
Never married	59.6	48.1	58.6	53.5	55.2
Married	11.7	13.4	8.3	10.7	10.7
Separated	7.6	7.5	10.5	10.7	9.2
Divorced	10.5	19.8	16.5	17.1	16.2
Widowed	10.5	11.2	6.0	8.0	8.6
Pregnant	6.1	1.1	1.9	7.5	3.9
In prenatal care (if pregnant)	40.0	100	60.0	38.5	46.7
Injection drug use	1.8	8.1	1.9	2.1	3.3
Time since first HIV positive test					
Within the last year	1.8	3.7	4.9	1.6	3.2
1–2 years	1.8	3.7	3.4	4.3	3.6
3–5 years	4.1	8.0	11.0	9.7	8.6
6–10 years	15.8	16.0	15.2	18.9	16.4
>10 years	76.6	68.5	65.5	65.4	68.5

**Table 3.** HIV Knowledge, Attitudes, and Beliefs and HIV risk behaviors by follow-up time point, CMEP-WILLOW, November 2011 - February 2015.

	Baseline %	Follow-up 1		Follow-up 2	
	
		%	aPR (95% Cl)	%	aPR (95% Cl)

Knowledge, Attitudes, and Beliefs^†^, %					
Knowledge about HIV (≥80% correct)	66.0	79.5	1.19 (1.13–1.26)***	81.2	1.23 (1.16–1.30)***
Attitudes and beliefs toward condom use (partner)^‡^	84.3	89.2	1.05 (1.02–1.09)**	88.6	1.05 (1.01–1.09)**
Attitudes and beliefs toward condom use (self)^‡^	79.5	85.3	1.08 (1.03–1.12)***	83.6	1.05 (1.01–1.09)*
Ability to discuss condom use^‡^	71.3	80.9	1.13 (1.07–1.19)***	79.9	1.12 (1.06–1.18)***
Ability to use condoms^‡^	68.9	85.8	1.24(1.18–1.30)***	85.3	1.23 (1.17–1.29)***
Adherence to ART^‡^	93.2	N/A	NA	96.1	1.03 (1.01–1.06)*
Sex without a condom^§^					
Any sex events without a condom, %	28.7	24.0	0.83 (0.68–1.01)	19.3	0.69 (0.55–0.86)**
By partner attributes					
Primary	24.0	19.8	0.81 (0.65–1.01)	16.6	0.71 (0.55–0.91)**
Casual	5.9	4.3	0.73 (0.40–1.33)	3.4	0.61 (0.32–1.15)
HIV unknown	5.4	4.7	0.89 (0.50–1.57)	3.3	0.64 (0.33–1.25)
HIV negative	12.6	10.3	0.81 (0.58–1.14)	8.2	0.66 (0.46–0.96)*
Anonymous	1.2	0.6	NA	0.6	NA
PWID	1.7	0.9	NA	0.9	NA
Spent 24 hours in jail	2.9	2.9	NA	1.8	NA
Had an STI	1.0	0.0	NA	0.0	NA
Context of sex					
One-night stand	1.7	1.2	NA	0.9	NA
Exchange (received something)	2.5	1.2	NA	1.5	NA
Exchange (gave something)	0.5	0.3	NA	0.0	NA
While intoxicated or high on drugs	9.8	6.4	0.67 (0.44–1.01)	3.9	0.40 (0.24–0.67)***
	Mean %	Mean %	aRR (95% Cl)	Mean %	aRR (95% Cl)
% sex events that were without a condom	27.0	21.7	0.72 (0.56–0.92)**	18.2	0.57 (0.43–0.75)***

aPR = adjusted prevalence ratio; NA = Not applicable (medication adherence was only analyzed comparing follow-up 2 to baseline and some outcomes were too infrequently observed to analyze in models);

† *N* = 659;

‡ Above the 25th percentile;

§ *N* = 409, excluding individuals who reported no sex events at any time point; Analyzed using GEE with a Poisson distribution for dichotomous outcomes and a negative binomial model for the percent sex events that were unprotected, using the log-number of total events as an offset; Models adjusted for CBO, age, race/ethnicity, educational attainment, time since first testing positive for HIV, and reporting a primary partner;

* *p* < .05

** *p* < .01

*** *p* < .001.
