# Supplementary material for: Evaluation of an HIV prevention intervention for women living with HIV
Source: AIDS Care. Author manuscript; Available in PMC 2024 Mar 13. (PMC10935592; doi:10.1080/09540121.2019.1659910)
Supplement: KAB questions [file NIHMS1616049-supplement-KAB_questions.doc]

**CMEP-WILLOW – HIV Knowledge, Attitudes, and Beliefs Interview Questions**

**HIV Knowledge**

1. **You have to take a test to know if you have HIV.**

- True
- False
- Don’t know
- Decline to answer

1. **A positive HIV test means that you have AIDS.**

- True
- False
- Don’t know
- Decline to answer

1. **Condoms can help protect you from transmitting or becoming infected with HIV.**

- True
- False
- Don’t know
- Decline to answer

1. **Having sex with someone who has HIV is the only way to get HIV.**

- True
- False
- Don’t know
- Decline to answer

1. **It is important to use a condom if both you and your partner have HIV.**

- True
- False
- Don’t know
- Decline to answer

1. **There are different types of HIV infections.**

- True
- False
- Don’t know
- Decline to answer

1. **Female condoms can stop you from getting HIV.**

- True
- False
- Don’t know
- Decline to answer

1. **Having anal sex without a condom makes it easier to get HIV.**

- True
- False
- Don’t know
- Decline to answer

1. **Having a STD can make it easier to get HIV.**

- True
- False
- Don’t know
- Decline to answer

1. **Having a STD can make it harder to manage your HIV.**

- True
- False
- Don’t know
- Decline to answer

**Attitudes and Beliefs Toward Condoms for Partners**

1. **My partner would get mad if I said we had to use a condom.**

- Agree
- Disagree
- Decline to Answer

1. **My partner would think I was having sex with another person if I said we had to use a condom.**

- Agree
- Disagree
- Decline to Answer

**Attitudes and Beliefs Toward Condom Use for Self**

1. **Condoms ruin the mood.**

- Agree
- Disagree
- Decline to Answer

1. **Sex doesn’t feel as good when you use a condom.**

- Agree
- Disagree
- Decline to Answer

1. **Sex with male condoms doesn’t feel natural.**

- Agree
- Disagree
- Decline to Answer

1. **Using condoms breaks up the rhythm of sex.**

- Agree
- Disagree
- Decline to Answer

1. **Using condoms would help build trust between my partner and me.**

- Agree
- Disagree
- Decline to Answer

**Ability to Discuss Condom Use**

1. **Can you discuss condom use with your partner?**

- Yes
- Maybe
- No
- Decline to answer

1. **Can you insist on condom use if your partner does not want to use one?**

- Yes
- Maybe
- No
- Decline to answer

1. **Can you stop and look for condoms when you are sexually aroused?**

- Yes
- Maybe
- No
- Decline to answer

1. **Can you insist on condom use every time you have sex even when you are under the influence of drugs or alcohol?**

- Yes
- Maybe
- No
- Decline to answer

1. **Can you insist on condom use every time you have sex even when your partner is under the influence of drugs?**

- Yes
- Maybe
- No
- Decline to answer

1. **Can you insist on condom use every time you have sex even if you or your partner uses another method to prevent pregnancy?**

- Yes
- Maybe
- No
- Decline to answer

1. **Can you put a condom on your partner without spoiling the mood?**

- Yes
- Maybe
- No
- Decline to answer

**Ability to Use Condoms**

**HOW SURE ARE YOU THAT YOU COULD…**

1. **Put a condom on a hard penis.**

- Not at all
- Somewhat
- Very sure
- Decline to answer

1. **Unroll a condom down correctly on the first try.**

- Not at all
- Somewhat
- Very sure
- Decline to answer

1. **Start over with a new condom if you placed it on the wrong way.**

- Not at all
- Somewhat
- Very sure
- Decline to answer

1. **Unroll a condom fully to the base of the penis.**

- Not at all
- Somewhat
- Very sure
- Decline to answer

1. **Squeeze air from the tip of a condom.**

- Not at all
- Somewhat
- Very sure
- Decline to answer

1. **Take a male condom off without spilling the semen or cum.**

- Not at all
- Somewhat
- Very sure
- Decline to answer

1. **Take a male condom off before your partner loses their hard on.**

- Not at all
- Somewhat
- Very sure
- Decline to answer

1. **Dispose of a used condom properly.**

- Not at all
- Somewhat
- Very sure
- Decline to answer

1. **Use lubricant with a condom.**

- Not at all
- Somewhat
- Very sure
- Decline to answer
